# 

**Published:** 1897-11-13

**Authors:** 


					THE HOSPITAL. ABSOLUTELY PURE, therefore BEST. Nov. 13th, 1897.
CADBURY'S t0^k ww CADBURY'S
oocoa 'HIP IhI cocoa
no alkalies used.
a-
No. 581, Vol. XXIII. Satoeday,
Nov. 13th, 1897.
[Subscription TermB,] pRICE TWOPENCE.

				

